# Zika Virus Infection at Different Pregnancy Stages: Anatomopathological Findings, Target Cells and Viral Persistence in Placental Tissues

**DOI:** 10.3389/fmicb.2018.02266

**Published:** 2018-09-25

**Authors:** Lucia de Noronha, Camila Zanluca, Marion Burger, Andreia Akemi Suzukawa, Marina Azevedo, Patricia Z. Rebutini, Iolanda Maria Novadzki, Laurina Setsuko Tanabe, Mayra Marinho Presibella, Claudia Nunes Duarte dos Santos

**Affiliations:** ^1^Laboratório de Patologia Experimental, Pontifícia Universidade Católica do Paraná, Curitiba, Brazil; ^2^Laboratório de Virologia Molecular, Instituto Carlos Chagas, Fundação Oswaldo Cruz, Curitiba, Brazil; ^3^Secretaria da Saúde do Estado do Paraná, Curitiba, Brazil; ^4^Laboratório Central do Estado do Paraná, Curitiba, Brazil

**Keywords:** Zika virus, vertical transmission, placenta, anatomopathological analysis, Hofbauer cells, morphometric analysis

## Abstract

Zika virus (ZIKV) infection in humans has been associated with congenital malformations and other neurological disorders, such as Guillain-Barré syndrome. The mechanism(s) of ZIKV intrauterine transmission, the cell types involved, the most vulnerable period of pregnancy for severe outcomes from infection and other physiopathological aspects are not completely elucidated. In this study, we analyzed placental samples obtained at the time of delivery from a group of 24 women diagnosed with ZIKV infection during the first, second or third trimesters of pregnancy. Villous immaturity was the main histological finding in the placental tissues, although placentas without alterations were also frequently observed. Significant enhancement of the number of syncytial sprouts was observed in the placentas of women infected during the third trimester, indicating the development of placental abnormalities after ZIKV infection. Hyperplasia of Hofbauer cells (HCs) was also observed in these third-trimester placental tissues, and remarkably, HCs were the only ZIKV-positive fetal cells found in the placentas studied that persisted until birth, as revealed by immunohistochemical (IHC) analysis. Thirty-three percent of women infected during pregnancy delivered infants with congenital abnormalities, although no pattern correlating the gestational stage at infection, the IHC positivity of HCs in placental tissues and the presence of congenital malformations at birth was observed. Placental tissue analysis enabled us to confirm maternal ZIKV infection in cases where serum from the acute infection phase was not available, which reinforces the importance of this technique in identifying possible causal factors of birth defects. The results we observed in the samples from naturally infected pregnant women may contribute to the understanding of some aspects of the pathophysiology of ZIKV.

## Introduction

Zika virus (ZIKV) is an emergent arthropod-borne virus that belongs to the genus *Flavivirus* of the *Flaviviridae* family [International Comittee on Taxonomy of Viruses (ICTV), 2017]. This virus is primarily transmitted through the bite of the *Aedes* mosquito (Zanluca and Duarte dos Santos, 2016). Unlike most other flaviviruses, however, person-to-person ZIKV transmission is possible, although the contribution of this transmission mode to maintaining an epidemic is unclear. Transmission by sexual and perinatal interactions and from blood and platelet transfusions has been described (Mlakar et al., 2016; Noronha et al., 2016; Miner and Diamond, 2017).

In general, ZIKV infection in humans is characterized as a self-limiting disease, and the most frequent signs and symptoms are low fever, myalgia, rash, arthralgia, headache and conjunctival hyperemia (Duffy et al., 2009; Zanluca et al., 2015). Nevertheless, cases of neurological manifestations, such as Guillain-Barré syndrome (Beckham et al., 2016; Noronha et al., 2016; Schuler-Faccini et al., 2016), have been reported in patients diagnosed with ZIKV. In addition, ZIKV infection during pregnancy has been associated with fetal malformations. Brain microcalcification and other central nervous system disorders, ocular abnormalities, and arthrogryposis are all a part of congenital Zika syndrome (Brasil et al., 2016; Melo et al., 2016; Schuler-Faccini et al., 2016). By March 17, 2017, thirty-one countries or territories in the Americas had reported central nervous system malformations that were potentially associated with ZIKV infection, and Brazil is the most affected country to date [World Health Organization (WHO), 2017].

Since June 2015, we have been receiving samples of serum, urine and other body fluids for ZIKV diagnosis. Additionally, during the peak of the ZIKV outbreak in Brazil, in agreement with the local health authorities, most pregnant women in Paraná State suspected of having ZIKV infection were monitored. Samples of tissues, such as the placenta and umbilical cord, as well as fetal tissues (in the case of stillbirths), all of which were collected at the time of delivery, were sent to our laboratory for analysis.

Here, we present a case series in which we analyzed placental tissues from women infected with ZIKV at different pregnancy stages, focusing on the anatomopathological and morphometric findings, target cells and viral persistence.

## Materials and methods

### Patients and ethics approval

This study was approved by Fiocruz and the Brazilian National Ethics Committee of Human Experimentation under the number CAAE: 42481115.7.0000.5248. Since our laboratory is a Reference Center for the Diagnosis of Emerging Viruses of the Brazilian Ministry of Health, we obtained the waiver for the written informed consent to work with these samples. We are in compliance we all the ethical principles and our study was approved by the above-mentioned Ethical Committee.

During the ZIKV outbreak in Brazil (2015–2016), 331 pregnant women exhibiting ZIKV infection-compatible symptoms presented to the Public Health Units in Paraná State. Two hundred ninety-two of them tested negative for ZIKV infection, and 39 cases were laboratory-confirmed for ZIKV by RT-PCR. From the positive cases, two patients had spontaneous abortions, and the remaining 37 women were monitored during pregnancy until the time of delivery.

This report describes the anatomopathological and morphometric findings from the placental tissues of 24 pregnant women with confirmed ZIKV infection, i.e., those with positive results by RT-qPCR or immunohistochemical (IHC) analysis of placental tissue.

Although the ZIKV infection had occurred at different gestational periods, the fragments of all but one placental tissue analyzed in this study were collected immediately after delivery and frozen. The presence of the ZIKV genome in the samples was investigated by RT-qPCR, and when appropriate, the presence of antibodies in the serum samples was screened with an ELISA, as described below. An additional fragment of the placenta was kept in buffered formalin for further anatomopathological, morphometric and IHC analyses. The clinical condition of the newborns was evaluated immediately after delivery (no follow-up was performed).

### RNA extraction and viral genome detection

Viral RNA was isolated from 140 μL of biological fluids (serum, cerebrospinal fluid, lacrimal fluid, saliva or urine) using a QIAamp Viral RNA Mini Kit (Qiagen, Hilden, Germany) according to the manufacturer's protocol. RNA extraction from unfixed placenta or viscera was performed with a RNeasy Mini Kit (Qiagen), while RNA from formalin-fixed paraffin-embedded (FFPE) tissues was extracted using the ReliaPrep™ FFPE Total RNA Miniprep System (Promega, Madison, USA). RNA was stored at −80°C until use.

The primers and probes were synthesized and purified by Integrated DNA Technologies (IDT, Coralville, USA). The reporter dye 5-FAM was used for the probes. All real-time assays were performed with the GoTaq Probe 1-Step RT-qPCR System (Promega) with amplification by a LightCycler 96 instrument (Roche, Mannheim, Germany).

The ZIKV genome was detected according to the protocol of Lanciotti et al. (Lanciotti et al., 2008), which used 5 μL of RNA per 20 μL reaction, while the dengue virus (DENV) serotypes were detected according to the protocol of Johnson et al. (Johnson et al., 2005). Human RNAse P was used as an endogenous control (Emery et al., 2004).

Positive controls for ZIKV and DENV were obtained by virus isolation from Brazilian patients' sera in C6/36 or Vero E6 cells.

### Antibody detection

Anti-ZIKV IgM in serum samples was detected with an in-house IgM capture enzyme-linked immunosorbent assay (MAC-ELISA) using β-propiolactone-inactivated ZIKV and MOCK (from noninfected cells) cell cultures as antigens according to the Centers for Disease Control and Prevention [Centers for Disease Control Prevention (CDC), 2017] guidelines, with minor modifications. A humanized anti-flavivirus monoclonal antibody (MAb) was kindly provided by the CDC and was used as a positive control. Serum samples were also tested for anti-DENV antibodies using either the Panbio Dengue IgM Capture ELISA or the Panbio Dengue IgG Indirect ELISA (Alere, Brisbane, Australia) following the manufacturer's instructions.

### Anatomopathological, immunohistochemical and morphometric analysis

Placentas were fixed in 10% buffered formalin immediately after delivery.

Umbilical cord, membranes, and disk were examined. Multiple parallel sections were taken through the disk at 2-cm intervals.

Histologic samples of placental villous tissue were made from separate areas of distinct cotyledons. At least three sections were taken from the parenchyma in such a way that both chorionic and basal plates were included. Also, sections from the cord and the “jelly roll” of membranes were examined.

Deparaffinization and rehydration of the FFPE placental samples were performed using xylene and ethanol baths, respectively, and hydrogen peroxide/methanol was used to block endogenous peroxidase activity. Then, the sections were incubated with anti-flavivirus (4G2) or specific anti-ZIKV (produced at ICC/Fiocruz-PR) MAb and then with rabbit anti-mouse secondary antibody followed by goat anti-rabbit antibody conjugated to HRP (Spring Bioscience, Pleasanton, USA). Finally, the slides were incubated with a freshly prepared substrate mixture (DAB, DakoCytomation) and counterstained with Mayer's hematoxylin.

The anti-ZIKV MAb did not show any cross reactivity with other flaviviruses (dengue serotypes 1–4 and the yellow fever, Saint Louis encephalitis and West Nile viruses) in a cell culture model (Supplementary Figure 1). Additionally, 73 placental samples of other etiologies were tested by IHC using anti-ZIKV MAb, and no cross reactivity was observed. Also, RT-qPCR yielded negative results in most of these samples.

Negative controls were obtained either by omitting the primary antibody from the incubation step or by using an unrelated MAb against Chikungunya virus (CHIKV), produced at ICC/Fiocruz-PR, as the primary antibody (Supplementary Figure 2).

To better identify Hofbauer cells (HCs), the FFPE placental tissue specimens were incubated with the primary antibody anti-human CD163 (Invitrogen, Carlsbad, USA) by using the same protocol.

The placenta slides (three slides from the placental disc, one slide from the chorioamniotic membrane and one slide from umbilical cord) were observed with an Olympus™ BX50 optical microscope (Tokyo, Japan), and photomicrographs were acquired in a high-power field (400×) using a Zeiss Axio Scan.Z1™ scanner (Carl Zeiss, Thornwood, USA) for both routine hematoxylin-eosin (H&E) staining and IHC staining. Villus maturation was evaluated by considering the villus morphology and the gestational age at delivery.

Morphometric analyses were performed in placental tissue specimens from mothers who were infected with ZIKV during the first (*n* = 4), second (*n* = 7), and third trimester of pregnancy (*n* = 6). A negative control group (*n* = 6) was used for comparison (Baurakiades et al., 2011). For each case, 30 high-power fields (HPFs = 400×) were randomly selected, and the knots and sprouts in each field were counted. The mean values of these parameters over the 30 fields were used for the statistical analyses. The Zika-infected and uninfected control groups were compared using the One-way ANOVA followed by Dunnett's multiple comparison test. Similarly, villi and HCs CD163^+^ cells were counted in 30 fields from placental samples from mothers infected during the third trimester of pregnancy (*n* = 5) and from a negative control group (*n* = 3). The Zika-infected and uninfected control groups were compared using the Mann Whitney test.

For both, statistical analyses were performed using GraphPad Prism 6.01 software. Differences were considered to be statistically significant at *p* < 0.05.

## Results

This report describes the evaluation of the 24 cases of ZIKV infection confirmed in pregnant women at different gestational periods (Table 1).

**Table 1:** Pathological features and laboratory findings from 24 cases of Zika virus infection confirmed during pregnancy.

TIME of infection–gestational trimester/Probable month of infection^*^		**Patient code**	**RT-PCR (serum)**	**RT-PCR (urine)**	**Anti-ZIKV IgM (serum)**	**Anti-DENV Serology**	**IHC Placenta (FFPE)**	**RT-PCR Placenta (FFPE)**	**RT-PCR Placenta (unfixed)**	**Gestational age/Pathological features**	**Congenital disorder**	**Outcome/Delivery gestational age**	**Newborn samples–additional observation**
First Trimester (weeks 1–13)	2nd	LRV/16 1350	+	–	I	IgM-/IgG-	–	NT	–	Third trimester/umbilical artery agenesis	Yes—microcephaly, ventriculomegaly, calcifications	Newborn/preterm (36 w)	CSF, urine, lacrimal fluid and saliva RT-PCR-
	3rd	LRV/16 1318	+	–	+	IgM-/IgG+	–	NT	–	Third trimester/without pathological changes	No	Newborn/term	CSF, Lacrimal fluid and saliva RT-PCR- Serum RT-PCR-/IgM-
	1st	LRV/15 578	NA	NA	NA	NA	–^†^	+	NA	Third trimester/villous immaturity	Yes—microcephaly, arthrogryposis, perinatal death	Newborn/term	Brain tissue RT-PCR+
	1st	LRV/16 1317	+	NA	+	IgM-/IgG+	–	NT	–	Third trimester/without pathological changes	No	Newborn/term	NA
	2nd	LRV/15 572	NA	NA	NA	NA	+	+	NA	First trimester/chronic villitis TORCH-like	No	Spontaneous abortion/12 w	NA
Second Trimester (weeks 14–26)	6th	LRV/16 870	+	NA	+	IgM-/IgG+	–	–	–	Third trimester/without pathological changes	No	Newborn/term	NA
	4th	LRV/16 1065	+	–	+	IgM-/IgG+	–	NT	–	Third trimester/without pathological changes	NO	Newborn/term	Lacrimal fluid and saliva RT-PCR-. Serum RT-PCR-/IgM+ Colostrum IgM+
	5th	LRV/16 933	+	–	I	IgM-/IgG+	–	–	+	Third trimester/without pathological changes	No	Newborn/term	NA
	6th	LRV/16 1068	+	–	+	IgM-/IgG+	–	NT	+	Third trimester/mild acute funisitis	No	Newborn/term	Lacrimal fluid and saliva RT-PCR-
	6th	LRV/16 995	+	NA	I	IgM-/IgG-	–	–	–	Third trimester/without pathological changes	Yes—Spina bifida	Newborn/term	NA
	5th	LRV/16 1029	+	–	+	IgM-/IgG+	–	–	–	Third trimester/without pathological changes	No	Newborn/term	Lacrimal fluid, saliva, serum RT-PCR-/IgM- Colostrum IgM+
	5th	LRV/16 1004	+	–	+	IgM-/IgG+	–	–	+	Third trimester/without pathological changes	No	Newborn/term (40 w)	Serum RT-PCR-/IgM-
	6th	LRV/16 845	+	NA	–	IgM-/IgG-	+	–	–	Third trimester/villous immaturity	No	Newborn/term	NA
Third Trimester (weeks 27–40)	8th	LRV/16 927	NA	–	NA	NA	+	–	–	Third trimester/without pathological changes	No	Newborn/term	NA
	7th	LRV/16 854	+	NA	I	IgM-/IgG-	+	–	–	Third trimester/villous immaturity	No	Newborn/term	NA
	7th	LRV/16 859	–	–	+	IgM-/IgG+	+	–	–	Third trimester/without pathological changes	No	Newborn/term	NA
	7th	LRV/16 931	+	NA	I	IgM-/IgG-	–	–	–	Third trimester/without pathological changes	No	Newborn/term	NA
	7th	LRV/16 848	NA	NA	NA	NA	+	–	–	Third trimester/without pathological changes	No	Newborn/term	NA
	8th	LRV/16 284	–	+	+	IgM-/IgG+	–	NT	+	Third trimester/without pathological changes	No	Newborn	Serum RT-PCR-/IgM-
Unknown	?	LRV/16 857	NA	NA	NA	NA	+	–	–	Third trimester/without pathological changes	Yes—Hydrocephalus	Newborn/preterm (34 w)	NA
	?	LRV/16 103	NA	NA	NA	NA	+	–	–	Third trimester/villous immaturity	Yes—Stillborn	Intrauterine fetal demise/term	Viscera RT-PCR-
	?	LRV/16 515	NA	NA	NA	NA	+	–	–	Third trimester/villous immaturity	Yes—brain abnormalities, encephalocele, death at 1-month-old	Newborn/term (38 w)	Viscera and CSF RT-PCR-
	?	HC N16-09	NA	NA	NA	NA	+	–	NA	Third trimester/villous immaturity	Yes—Microcephaly, discrete multifocal cerebritis, calcifications	Intrauterine fetal demise/term	NA
	?	LRV/16 855	NA	–	NA	NA	+	–	–	Third trimester/without pathological changes	Yes—Hydrocephalus, ventriculomegaly, calcifications, bilateral congenital cataract	Newborn/preterm (34 w)	CSF RT-PCR-

* For term placentas, the probable month of infection was calculated assuming delivery at week 39 as described by Moore et al. (Moore et al., 2014).

† *IHC was positive in the brain tissue of the newborn. ?, not known; +, positive; −, negative; I, inconclusive; NT, not tested; NA, not available; w, weeks; CSF, cerebrospinal fluid*.

Serological analysis was performed in the 15 cases for which serum samples were available; nine of these cases presented with both anti-ZIKV IgM and anti-DENV IgG, and one had only anti-DENV IgG. The presence of these IgG antibodies suggests a past *Flavivirus* infection. None of the anti-ZIKV IgM-positive samples cross reacted in the anti-DENV IgM ELISA (Table 1, cases LRV/16 1318, 16 1317, 16 870, 16 1065, 16 1068, 16 1029, 16 1004, 16 859, and 16 284).

Neither ZIKV RNA nor anti-ZIKV IgM were detected in samples of amniotic fluid, newborn cerebrospinal fluid (CSF) or ocular/oral swabs in the cases described in this article. Of the five newborn serum samples available, one presented anti-ZIKV IgM, indicating transplacental infection, although no congenital disorder was observed at the time of delivery in this case (Table 1, case LRV/16 1065).

**Figure 1:**
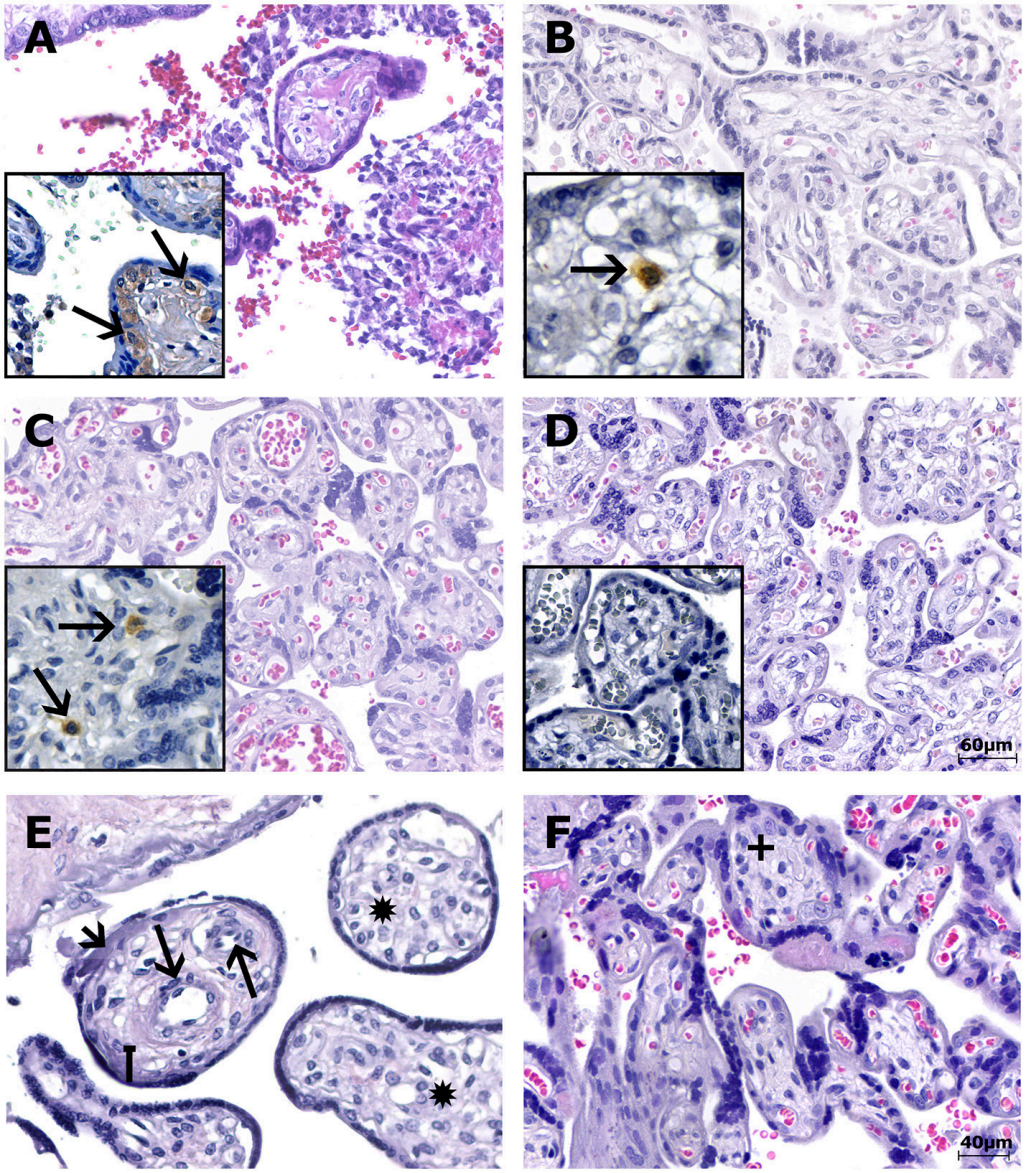
Photomicrography of the placental samples stained with H&E or immunostained with anti-ZIKV MAb and stained with Harris's hematoxylin (squares). The scale bars are 60 μm **(A–D)** and 40 μm **(E,F)**. **(A)** Case LRV/15 572—First trimester placenta sample (chorion frondosum) showing chronic villitis (TORCH-like) with lymphohistiocytic chronic villous inflammation. **(B)** Case LRV/16 515—Third trimester placenta sample (chorion frondosum) showing delayed villous maturation with additional stromal changes, such as stromal fibrosis. **(C)** Case LRV/16 845—Third trimester placenta sample (chorion frondosum) showing delayed villous maturation with persistence of the cytotrophoblastic layer, stromal fibrosis and reduced numbers of syncytial knots. **(A–C)** The squares highlight Hofbauer cells positive for anti-ZIKV MAb (arrows) inside the chorionic villi. Notice that the cytotrophoblast and syncytiotrophoblast cells, as well as other fibroblastic cells inside Wharton's jelly, are negative for anti-ZIKV MAb. **(D)** Negative control (ZIKV-negative patient)—Third trimester placenta sample (chorion frondosum) without pathological changes. Hofbauer cells are negative for anti-ZIKV MAb inside the chorionic villi (square). Note that the cytotrophoblast and syncytiotrophoblast cells, as well as other fibroblastic cells inside Wharton's jelly, are negative for the antibody used. **(E)** Case LRV/15 578—Third trimester placenta sample (chorion frondosum) showing delayed villous maturation with additional stromal changes, such as stromal fibrosis (square arrow), an increase in the number of fetal capillaries (arrows), hyperplasia of Hofbauer cells (^*^) and an increase in intravillous fibrinoid deposits (arrow head). **(F)** Negative control (ZIKV-negative patient)—Third trimester placenta sample (chorion frondosum) showing a normal number of Hofbauer cells (+). No delayed villous maturation or additional stromal changes were observed.

The anatomopathological findings for the FFPE placental tissue specimens are compiled in Tables 1, 2. Of note, although our panel includes samples from women who had ZIKV infection at different stages of pregnancy, all placental samples were obtained at the time of delivery except for one, which was from a spontaneous abortion at 12 weeks of gestation (first trimester placenta, case LRV/15 572). Additional information about this case has been published (Noronha et al., 2016). This ZIKV-positive first trimester human placental sample (LRV/15 572) exhibited chronic villitis and TORCH-like features that are shown in Table 2; Figure 1A.

**Table 2:** Main placental histopathological findings from infants with and without congenital disorder at birth.

**First trimester (*n* = 1)**	**Third trimester (*****n*** = **23)**	
Chronic villitis with TORCH-like features •Lymphohistiocytic chronic villous inflammation •Edema •Trophoblastic lesion •Increase in HCs •Increase in stromal lymphocytes •Histiocytes in the intervillous spaces	Without congenital disorder (*n* = 15) With Congenital Disorder (*n* = 8)	No pathological changes (*n* = 12) Delayed villous maturation with additional stromal changes^*^ (*n* = 2) Mild acute funisitis (*n* = 1) No pathological changes (*n* = 3) Delayed villous maturation with additional stromal changes^*^ (*n* = 4) Umbilical artery agenesis (*n* = 1)

HCs, Hofbauer cells.

* Stromal changes:

•*Persistence of the cytotrophoblastic layer*

•*Thickening of the trophoblastic basement membrane*

•*Stromal fibrosis*

•*Increase in the number of fetal capillaries*

•*Edematous or sclerotic villi*

•*Coarse calcification foci*

•*Hyperplasia of HCs*

•*Moderate increases in intravillous and perivillous fibrinoid deposits*.

Eight of the 23 third trimester human placental tissue samples exhibited pathological features in routine H&E-stained sections (whereas placentas without pathological signs were observed in the remaining 15 cases). The most prevalent alteration was delayed villous maturation with additional stromal changes, such as hyperplasia of HCs (75%), as shown in Table 2; Figures 1B,C,E. Of the nine cases exhibiting pathological signs in the H&E staining, five were associated with congenital disorders (55.6%), and one evolved to a spontaneous abortion (first trimester placenta, case LRV/15 572). In the remaining 15 cases, no pathological evidence was observed in the H&E sections, but three of these cases presented with congenital disorders (20%; Tables 1, 2). Of the six cases with villous immaturity, four had a congenital disorder diagnosed at birth (66.7%), two had intrauterine fetal death (Table 1), one had acute funisitis, and one had umbilical artery agenesis (Table 2).

**Figure 2:**
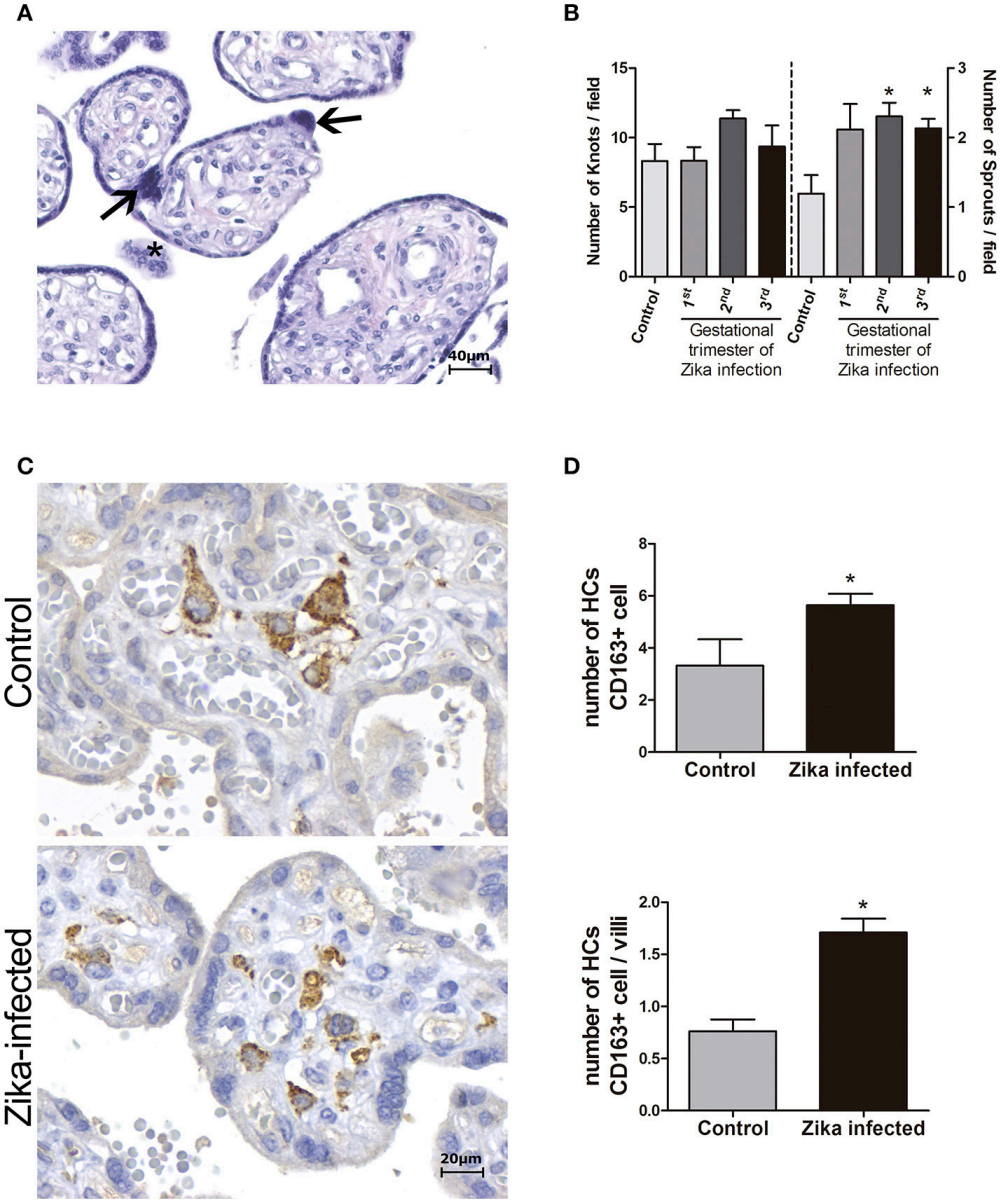
Morphometric analysis of placental specimens from women infected with ZIKV during the pregnancy and from negative controls. **(A)** Photomicrography of a placental sample stained with H&E showing syncytial knots (arrows) and sprouts (^*^). **(B)** Quantification of knots and sprouts. A significant increase in the number of placental sprouts was observed in the groups whose mothers were infected with ZIKV during the first (*n* = 4), second (*n* = 7), or third (*n* = 6) trimester of pregnancy, when compared to the negative controls (*n* = 6). **(C)** Immunostaining with CD163 highlights Hofbauer cell hyperplasia in ZIKV-infected placentas. **(D)** Quantification of CD163^+^ Hofbauer cells. The average numbers of CD163^+^ cells and CD163^+^ cells per villus were significantly higher in the ZIKV-positive group (*n* = 5) than in the negative controls (*n* = 3). **(B,D)** Thirty high-power fields (HPFs = 400×) for each case were randomly selected for counting. The mean of the 30 fields was used for the statistical analyses. The Zika-infected and negative control groups were compared using either the One-way ANOVA followed by Dunnett's multiple comparison test **(B)** or the Mann Whitney test **(D)**. The asterisks indicate statistically significant differences between the groups (^*^p < 0.05).

Morphometric analysis of the placentas from women infected during the third trimester of pregnancy confirmed the villous immaturity and hyperplasia of HCs (Figure 2). Syncytial knots and sprouts were quantified to demonstrate disorders of villus maturation, and a higher number of sprouts was detected in the ZIKV-infected group than in the control group (*p* < 0.05), regardless the gestational period of infection (Figure 2B). Villi and CD163^+^ HCs were measured to evaluate hyperplasia of HCs. Compared to the placentas in the control group, the placentas from women infected with ZIKV during the third trimester showed increased numbers of HCs (hyperplasia; Figures 2C,D).

**Figure 3:**
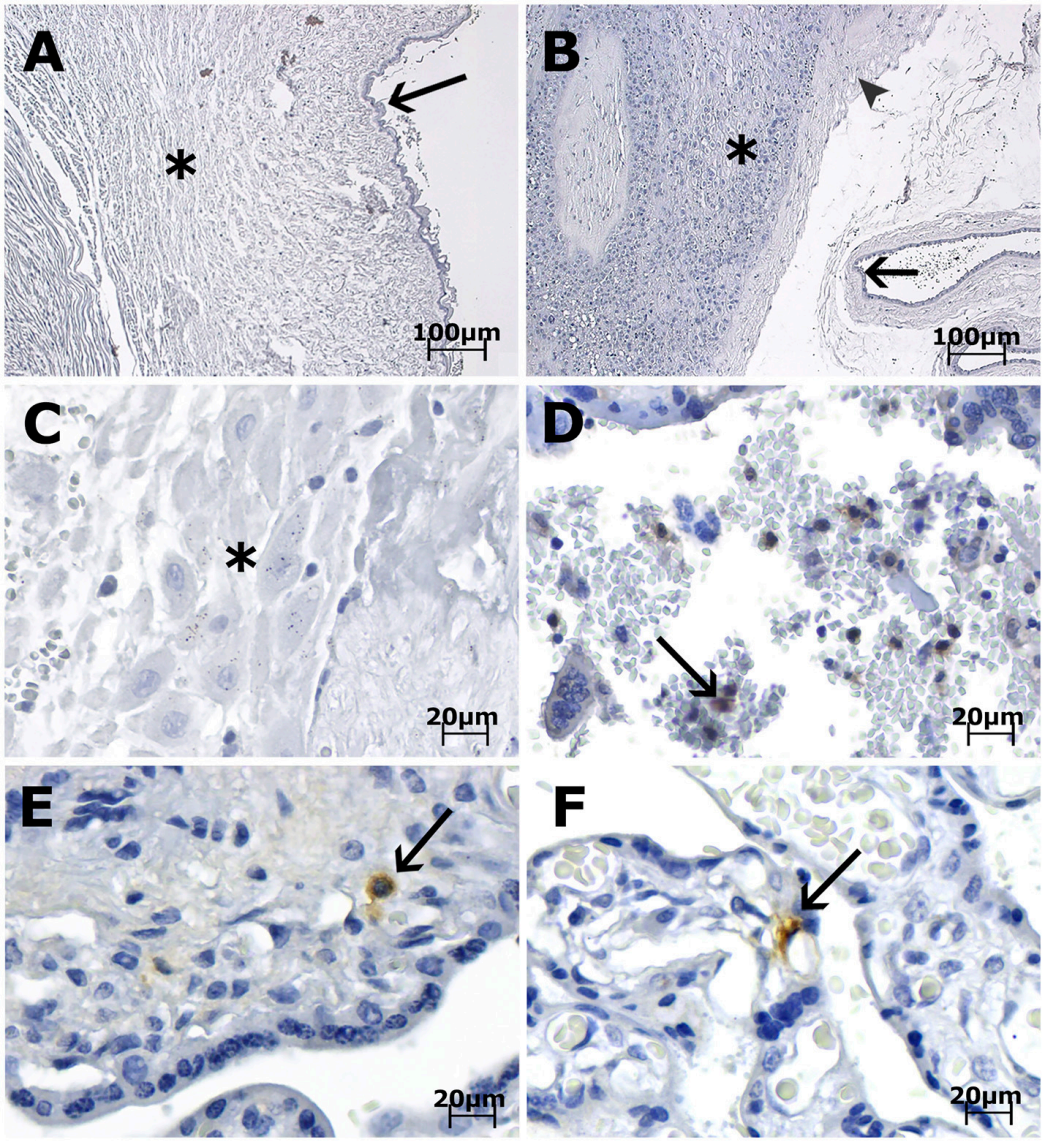
Photomicrography of placenta, cord and membrane samples from women diagnosed as positive for ZIKV infection immunostained with anti-ZIKV MAb and stained with Harris's hematoxylin. The scale bars are 100 μm **(A,B)** and 20 μm **(C–F)**. **(A)** Case LRV/16 515—Umbilical cord slides from a third trimester placenta negative for anti-ZIKV MAb. Fibroblasts in Wharton's jelly (^*^) and amniotic epithelium (arrow) are negative for the antibody used. **(B)** Case LRV/16 515—Chorioamniotic membrane from a third trimester placenta negative for anti-ZIKV MAb. Notice that the capsular decidua (^*^), the smooth chorion above the capsular decidua (arrowhead) and the amniotic epithelium (arrow) are negative for the antibody used. **(C)** Case LRV/16 515—Decidua basalis (^*^) from a third trimester placenta negative for anti-ZIKV MAb. **(D)** Case LRV/15 572—Intervillous space from a first trimester placenta with inflammatory cell infiltrates positive for anti-ZIKV MAb (arrow). **(E)** Case LRV/16 927 and **(F)** Case LRV/16 848: Chorion frondosum from third trimester placentas showing Hofbauer cells positive for anti-ZIKV MAb (arrows) inside the chorionic villi. Notice that the cytotrophoblast and syncytiotrophoblast cells, as well as fibroblastic cells inside Wharton's jelly, are negative for the antibody used.

Immunohistochemical (IHC) analysis of the placental tissue samples using anti-flavivirus MAb (4G2) and anti-ZIKV MAb showed immunostaining in the HCs, regardless of the gestational age when ZIKV infection occurred (Figures 1A–C, 3E,F). The cytotrophoblast (CTB) and syncytiotrophoblast (STB) cells (chorion frondosum), as well as the fibroblastic cells and Wharton's jelly, were negative in all specimens (Figures 1, 3E,F). All umbilical cord (fibroblasts, Wharton's jelly, and amniotic epithelium), chorioamniotic membrane (capsular decidua, amniotic epithelium, and smooth chorion above the capsular decidua), and decidua basalis samples were consistently negative for the anti-ZIKV and anti-flavivirus monoclonal antibodies (Figures 3A–C). The intervillous space showed maternal inflammatory cell infiltrates that were positive for both the anti-flavivirus and anti-ZIKV MAbs in only the first trimester placental sample (LRV/15 572) with villitis (Figure 3D). ZIKV infection of this sample was confirmed by RT-qPCR using RNA extracted from a FFPE tissue core corresponding to a villitis area (Noronha et al., 2016).

## Discussion

In this study, we examined human placental samples from naturally infected women, focusing on the anatomopathological and morphological aspects of the samples and on determining which cells were targeted by ZIKV during pregnancy.

The main histological findings (from H&E sections) in the placental tissues from women that were infected with ZIKV at different gestational periods were villous immaturity and stromal changes, such as hyperplasia of HCs, stromal fibrosis, edema of stromal villi, sclerotic villi, calcification foci and fibrinoid deposits (Table 2). Although studies on naturally infected placental tissues are limited, two previous reports corroborate our findings (Martines et al., 2016b; Ritter et al., 2017). Both reports detected chorionic villi with calcification, fibrosis, perivillous fibrin deposition, patchy intervillositis and focal villitis in the cases that presented pathological signs. Conversely, tissues from several ZIKV-positive samples had normal-appearing chorionic villi, in agreement with the results shown in Table 2 (Martines et al., 2016b; Ritter et al., 2017).

Morphometric analysis showed enhancement of the number of HCs and syncytial sprouts (indicating a villous maturation disorder) in the placentas of women infected with ZIKV at late gestational periods compared to the numbers of these features in the control group placentas. Syncytial knots are specializations in the syncytiotrophoblast, and an increase in these structures in late gestation indicates placental pathology and can be used to evaluate villous maturity. Syncytial sprouts are markers of trophoblast proliferation; they are seen frequently during early pregnancy and are increased in many diseases (Loukeris et al., 2010). These findings agree with the results of a study showing the damaging effect of type I interferon (INF I) on *ex vivo* human mid-gestation placental tissues treated with INF-β. The authors demonstrated that type I INFs trigger fetal death in a mouse model and that treatment of *ex vivo* human placentas with INF-β induces morphological changes in the villi, such as syncytial knots and sprouts (Yockey et al., 2018).

Immunohistochemical analysis revealed that HCs were the only ZIKV-positive fetal cells in the naturally infected human placental samples examined in this study, regardless of the gestational period at which infection occurred, and that HCs remain persistently infected until the time of delivery. It is noteworthy that even in the tissue samples from women infected in late pregnancy, i.e., with a short interval between the acute phase of infection and delivery time (LRV/16 854, 16 859, 16 931, 16 848, 16 927, and 16 284), ZIKV was detected exclusively in HCs (Table 1).

HCs are placental villous macrophages of fetal origin, and alterations in their numbers (hyperplasia) and biological features are associated with complications in pregnancy. HCs play a role in diverse functions, such as placental vasculogenesis, immune regulation and the secretion of enzymes and cytokines across the maternal-fetal barrier. In addition, the results from a double-labeling assay showed the association of Sprouty proteins (implicated in villus branch morphogenesis) with HCs, suggesting the involvement of HCs in the development of placental villi (Anteby et al., 2005).

HCs are categorized as placental M2 macrophages, and their location and migratory behavior confer an ability to move around the villous stroma and to make transient contacts with other macrophages and villous core cells (Khan et al., 2000; Tang et al., 2011).

Another fact worth mentioning is the apparent correlation between villous immaturity and congenital disorders caused by ZIKV infection since we observed the occurrence of fetal malformations in four out of six cases (66.7%) of villous immaturity (observed in H&E staining and morphometric analysis). Furthermore, villous immaturity may be related to an increase in HCs, the cells that sustain the presence of ZIKV in the placenta. These data could be valuable to pathologists and neonatologists following up on newborns exposed to ZIKV by vertical transmission.

Of note, the case of the first trimester placenta with villitis (LRV/15 572) exhibited ZIKV-positive maternal inflammatory cells in the intervillous space. Although positivity in fetal endothelium and maternal leukocytes (Ritter et al., 2017), and in decidual cells, CTBs and mesenchymal cells of chorionic villi (Rabelo et al., 2018) in a few first trimester placentas has also been reported, we speculate that maternal or other fetal cells may be infected only transiently. In support of this hypothesis, studies of natural ZIKV infections using either IHC or *in situ* hybridization analysis (ISH) have also reported that HCs were the most frequently observed infected cells (Martines et al., 2016a; Noronha et al., 2016; Bhatnagar et al., 2017; Rosenberg et al., 2017).

The persistence of ZIKV-positive fetal HCs in full-term placentas regardless of the period at which infection occurred indicates that ZIKV can persist in the placenta for several months after maternal infection and may provide a viral source for continued fetal infection.

Examination of the entire human placenta, comprising the umbilical cord, amniotic membrane, chorion frondosum (CTB and STB), smooth chorion, capsular decidua, and decidua basalis, revealed that all these tissues were consistently negative for ZIKV infection. Mlakar et al. (Mlakar et al., 2016) reported similar findings in a single case. Rabelo et al. (Rabelo et al., 2017) showed ZIKV NS1 protein in the decidual and endothelial cells of the maternal decidua and in CTB, STB, and HCs in the third trimester placental tissues associated with an HIV-exposed but uninfected infant with severe congenital Zika syndrome. Nonetheless, the maternal HIV infection could have contributed to the permissiveness of other maternal/placental cell types to ZIKV infection.

Thus, we suggest that the most plausible hypothesis for the transplacental transmission of ZIKV would be related to its association with HCs and its migratory ability to reach the fetal vessels and then infect the fetus either by transcytosis or through ZIKV-infected “Trojan horse” cells (Zanluca et al., 2018).

Furthermore, as both ZIKV genomic material and viral particles are detected in placental cells until the end of pregnancy (regardless of the trimester in which infection occurred), it is plausible to speculate that the infection of the fetus could happen as a secondary event, i.e., not necessarily concomitant with the maternal acute phase of disease (Aagaard et al., 2017). In this case, understanding the biology of HCs after ZIKV infection is of utmost importance in explaining the different congenital outcomes related to ZIKV infection (Simoni et al., 2017).

We emphasize that a negative ZIKV detection (by RT-PCR or IHC) in a placental sample does not exclude the possibility of maternal ZIKV infection. Possible reasons for false negative results include ZIKV RNA/protein levels below the limit of detection of the employed assays, RNA degradation due to storage/shipping processes or variability in tissue sampling, and the viral strain. For example, eight of the pregnant women described in Table 1 had ZIKV infection confirmed by RT-qPCR in the serum sample during the acute phase of infection, but no viral RNA/protein was detected in their placental tissues.

Conversely, in this study, ZIKV was also detected in placental samples from nine women who had an onset of ZIKV clinical symptoms during the first, second or third trimester but gave birth to normal infants. Abnormalities in these infants may have been prevented because placental integrity limited viral spreading from mother to fetus. However, we cannot exclude fetal exposure to ZIKV, and it is noteworthy that, in some cases, abnormalities are only detected months after delivery (Aragao et al., 2017; Ventura et al., 2017). Serological examination and clinical follow-up of the newborns are required to confirm and define the diagnosis. Periodic monitoring of these infants may be helpful for the early recognition of future sequelae from congenital infection (Bhatnagar et al., 2017). In conclusion, tissue analysis provides the opportunity to confirm maternal/placental ZIKV infection, which may alert the practitioner to the potential for a congenital disorder in the child.

## Author contributions

LN, CZ, MB, and CD contributed to the study design. CZ, AA, and MA performed the experiments. PR performed the morphometric analyses. MB, IN, and LT participated in the patient follow-up. MP participated in sample identification/distribution. LN, CZ, AA, and CD analyzed the results and wrote the manuscript.

### Conflict of interest statement

The authors declare that the research was conducted in the absence of any commercial or financial relationships that could be construed as a potential conflict of interest.

## Supplementary material

Supplementary Figure 1 Characterization of the anti-ZIKV MAb by immunofluorescence. The MAb recognized ZIKV-infected C6/36 cells and showed no crossreactivity with DENV serotypes 1–4 or with the yellow fever (YFV), West Nile (WNV) and Saint Louis encephalitis (SLEV) viruses. No reaction was observed in the MOCK-infected cells. The pan-flavivirus MAb 4G2 was used as the positive control, and an unrelated MAb was used as the negative control. The scale bars are 250 μm.

Supplementary Figure 2 Photomicrography of third trimester placental samples (chorion frondosum) from women diagnosed positive for ZIKV infection immunostained with anti-ZIKV **(A)**, anti-pan-flavivirus 4G2 **(B)** and anti-CHIKV MAbs **(C)** or with no primary antibody **(D)** and stained with Harris's hematoxylin. **(A,B)** The arrows indicate positive Hofbauer cells inside the chorionic villi. Notice that the cytotrophoblast and syncytiotrophoblast cells, as well as fibroblastic cells inside Wharton's jelly, are negative for both antibodies used. **(C,D)** No reaction was observed in the negative controls. The scale bars are 60 μm.
