# Supplementary figures and images for: Zika Virus Infection at Different Pregnancy Stages: Anatomopathological Findings, Target Cells and Viral Persistence in Placental Tissues
Source: Front Microbiol. 2018 Sep 25;9:2266. doi: 10.3389/fmicb.2018.02266 (PMC6180237; doi:10.3389/fmicb.2018.02266)

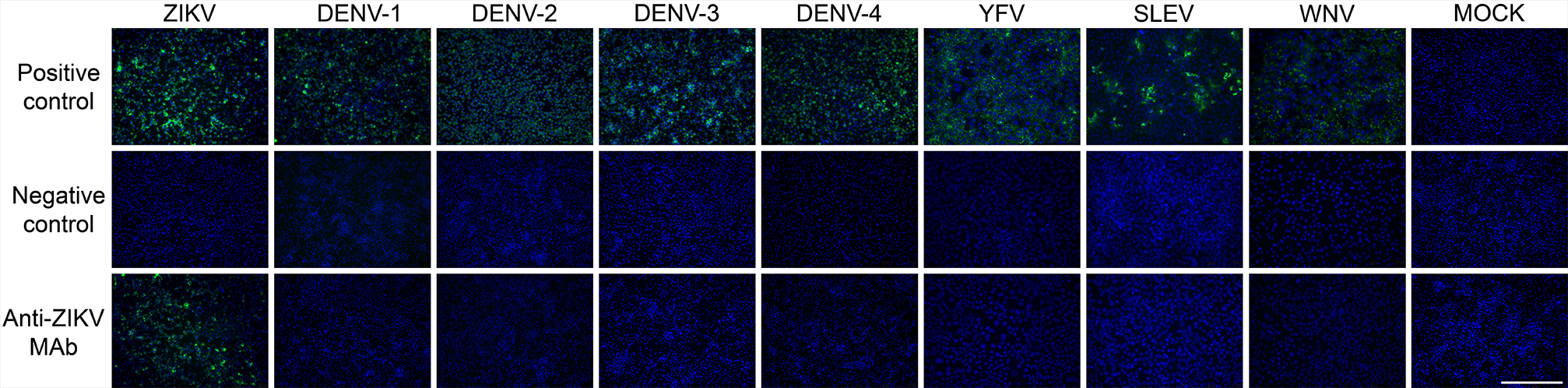

Supplement: Supplementary Figure 1 — Characterization of the anti-ZIKV MAb by immunofluorescence. The MAb recognized ZIKV-infected C6/36 cells and showed no crossreactivity with DENV serotypes 1–4 or with the yellow fever (YFV), West Nile (WNV) and Saint Louis encephalitis (SLEV) viruses. No reaction was observed in the MOCK-infected cells. The pan-flavivirus MAb 4G2 was used as the positive control, and an unrelated MAb was used as the negative control. The scale bars are 250 μm. [file Image_1.TIFF]

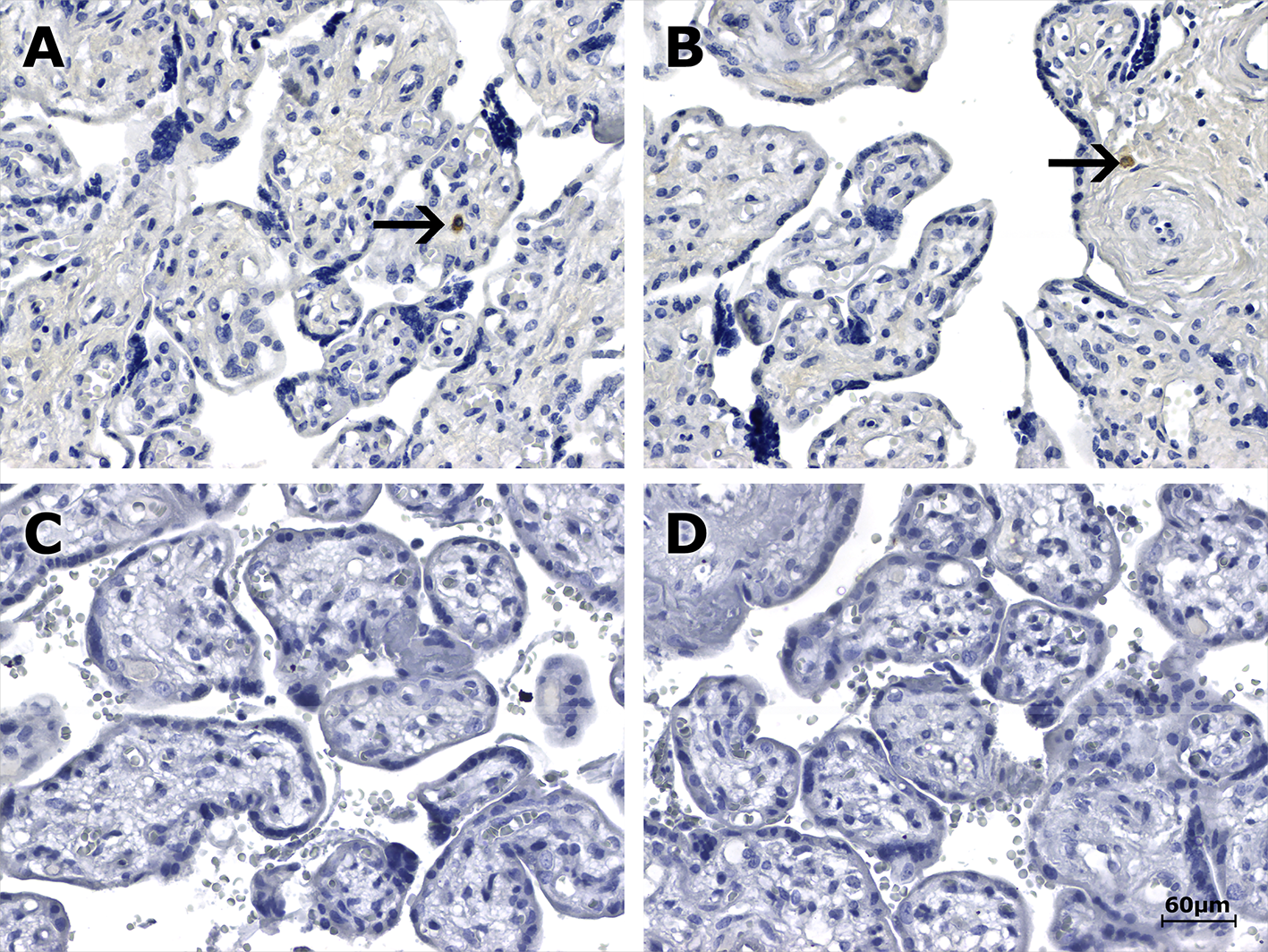

Supplement: Supplementary Figure 2 — Photomicrography of third trimester placental samples (chorion frondosum) from women diagnosed positive for ZIKV infection immunostained with anti-ZIKV (A), anti-pan-flavivirus 4G2 (B) and anti-CHIKV MAbs (C) or with no primary antibody (D) and stained with Harris's hematoxylin. (A,B) The arrows indicate positive Hofbauer cells inside the chorionic villi. Notice that the cytotrophoblast and syncytiotrophoblast cells, as well as fibroblastic cells inside Wharton's jelly, are negative for both antibodies used. (C,D) No reaction was observed in the negative controls. The scale bars are 60 μm. [file Image_2.TIFF]
